# Quasi-Static and Low-Velocity Impact Response of 3D Printed Plates Using Bio-Inspired Tool Paths

**DOI:** 10.3390/biomimetics10030135

**Published:** 2025-02-24

**Authors:** Muhammed Kamrul Islam, Paul J. Hazell, Hongxu Wang, Juan P. Escobedo, Harun Chowdhury

**Affiliations:** 1 School of Engineering and Information Technology, The University of New South Wales, Canberra, ACT 2600, Australia; kamrul.cuetme@gmail.com (M.K.I.); p.hazell@unsw.edu.au (P.J.H.); hongxu.wang@unsw.edu.au (H.W.); j.escobedo-diaz@unsw.edu.au (J.P.E.); 2 Walpett Engineering, Queanbeyan, NSW 2620, Australia; 3 ABSCUBE Engineering & Education Services Pty Ltd., Melbourne, VIC 3175, Australia

**Keywords:** bio-inspiration, Bouligand structure, additive manufacturing, quasi-static load, low-velocity impact

## Abstract

The study of biomimetics allows for the creation of various structures inspired by nature. This work investigates the impact of using a bio-inspired tool path for manufacturing porous plates via 3D printing. The Bouligand (or plywood-like) structure is prevalent in several biological components. Structures that mimicked the Bouligand design concerning the tool path were printed and compared to uniform plates produced with a rectilinear pattern through mechanical testing. Quasi-static and dynamic tests were conducted on specimens with infill densities ranging from 25% to 100%. Results indicated that the Bouligand pattern displayed superior specific energy absorption at 75% infill density. This bio-inspired path pattern also provided excellent elongation during quasi-static and dynamic failure—the fracture pattern of the bio-inspired path adhered to the Bouligand structure. In contrast, brittle failure was demonstrated by the specimen with a rectilinear pattern at varying infill percentages, while the bio-inspired pattern enhanced the toughness of the polymer specimens.

## 1. Introduction

**Figure 1:**
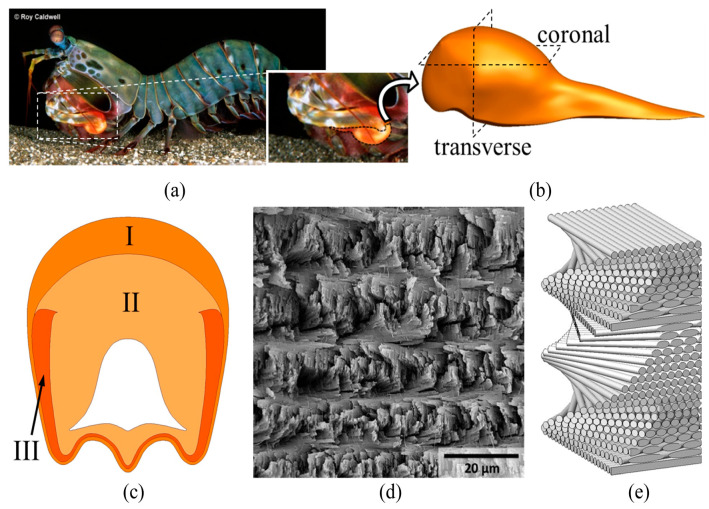
(**a**) Dactyl club of mantis shrimp (*Odontodactylus Scyllarus*), (**b**) 3D rendered image of dactyl club from CT scan, (**c**) Impact (I), periodic (II), and striated (III) regions from the transverse section, (**d**) SEM image of the periodic zone, and (**e**) 3D illustration of the Bouligand structure from the periodic zone [28,29,30].

Energy-absorbing lightweight structures are always in demand in many engineering applications, such as automotive, aerospace, and military sectors. Consequently, researchers have been investigating different manufacturing techniques, material compositions, structural designs, composite layups, and bio-inspirations to enhance their performance in energy-absorbing applications [1,2]. Bio-inspired structures are currently employed in aerospace, military, structural, and automotive applications where damage due to impact load is common [3,4]. Thus, composite structures with improved strength are being developed and tested for such applications. Researchers have been looking at different approaches to improve the strength of conventional structures. The biomimetic approach has become popular among scholars as nature is the best place to learn about sustainable structures with enhanced strength [5,6,7]. This approach allows one to choose a natural part or process and imitate its protective strategy to develop a novel structure [4]. Recent studies show numerous biological elements have shown excellent impact resistance, such as nacre [8,9,10], turtle shells [11,12,13], fish skins [14,15], bird beaks [16,17], horns [18,19], shark teeth [20], and horse hooves [21,22]. The desired properties, such as energy absorption and damage tolerance, are achieved by utilizing various biomimetic structures such as lamellar, fiber reinforcement, Bouligand, and brick and mortar. Several impact-resistant applications such as LAST (Light-appliqué Armor System Technology) tiles, stab-resistant plates, circular disks, and ceramic structures were implemented with the help of bio-inspiration [4]. Bouligand structure is a naturally developed hierarchical arrangement of plates or fibers organized in a twisted pattern, as shown in Figure 1. It is found in many biological parts like fish scales, crab exoskeletons, scorpion claws, and mantis shrimp’s dactyl clubs. The dactyl clubs can produce high-velocity strikes on the prey’s body without damaging the dactyl clubs [23,24]. Consequently, Bouligand-type structures have attracted the attention of researchers interested in bio-inspiration for damage tolerance and impact-resistant applications [25,26]. It should be noted that their impact performance at different infill densities still needs to be investigated. Advanced manufacturing techniques like 3D printing can fabricate structures following a specific bio-inspired tool path with varying infill densities [27]. In this work, a 3D printing tool path is modified following a Bouligand structure pattern inspired by the mantis shrimp’s dactyl club (Figure 1).

Three-dimensional (3D)-printed plate specimens were prepared using rectilinear and bioinspired tool paths with different infill percentages. The quasi-static and impact tests were the most commonly used techniques for examining the energy absorption capacity and damage mechanism of polymers [31], and they were subsequently performed for all the specimens. Several studies were conducted where the infill density was varied with an increment of 20 to 25% [32,33]. However, no study has been conducted to investigate the effect of a wide range of quasi-static and low-velocity impact load analyses on infill density. Therefore, this aims to study the effect of infill percentage in the case of quasi-static and low-velocity impact loads ranging from 25% to 50%, 75%, and 100%. The results from this study will help to understand the load bearing and energy absorption capacity at different infill densities, which vary with the change in printing patterns for developing lightweight structures using additive manufacturing.

## 2. Materials and Methods

### 2.1. Sample Preparation

Polylactic acid (PLA) is a biodegradable polymer widely used in 3D printing due to its low melting point, low thermal coefficient, and less adherence to the printing surface. Improved mechanical characteristics can be achieved using the proper extrusion temperature and layer height [22]. A PLA filament of 1.75 mm average diameter has been used in the Creality Ender 3 (Shenzhen, China) printer. The printer has a single nozzle with a 0.4 mm diameter and follows the fused deposition modeling (FDM) method to manufacture samples. The manufactured specimen size is 60 × 60 × 10 mm. The properties of printed PLA are listed in Table 1 and the printing parameters are listed in Table 2.

**Figure 2:**
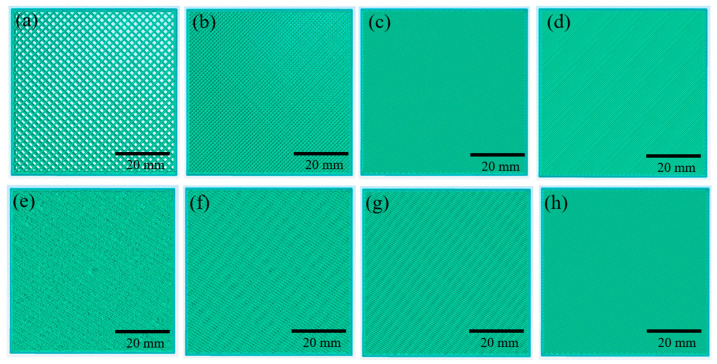
Rectilinear pattern (**a**) Infill 25%, (**b**) Infill 50%, (**c**) Infill 75%, (**d**) Infill 100%; Bouligand pattern (**e**) Infill 25%, (**f**) Infill 50%, (**g**) Infill 75%, (**h**) Infill 100%.

**Figure 3:**
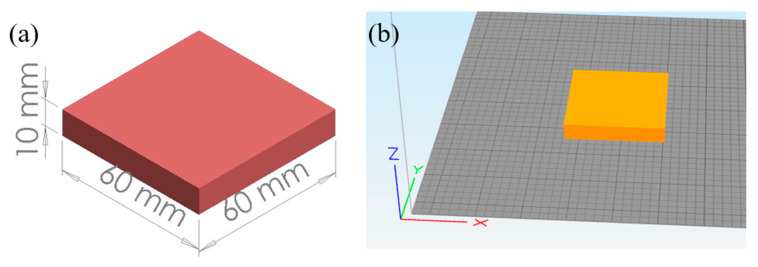
(**a**) Dimensions of the 3D-printed specimen, (**b**) Specimen setup for printing in Simplify3D.

Rectilinear and Bouligand printing patterns were set up in the Simplfy3D slicer software. The cross-sections of the patterns are illustrated in Figure 2. The rectilinear infill had an angle offset of +45°/−45°, whereas the Bouligand pattern had a 15° raster angle between two adjacent layers. The geometry of the specimens is illustrated in Figure 3. The outline shell thickness of the specimens was 0.8 mm. A summary of the fabricated structures is listed in Table 3. The specimen ID nomenclature is R_25P (or B_25P), where R represents the rectilinear tool path, B represents the Bouligand tool path, and 25P indicates that the infill density was 25%.

### 2.2. Quasi-Static Indentation Test

**Figure 4:**
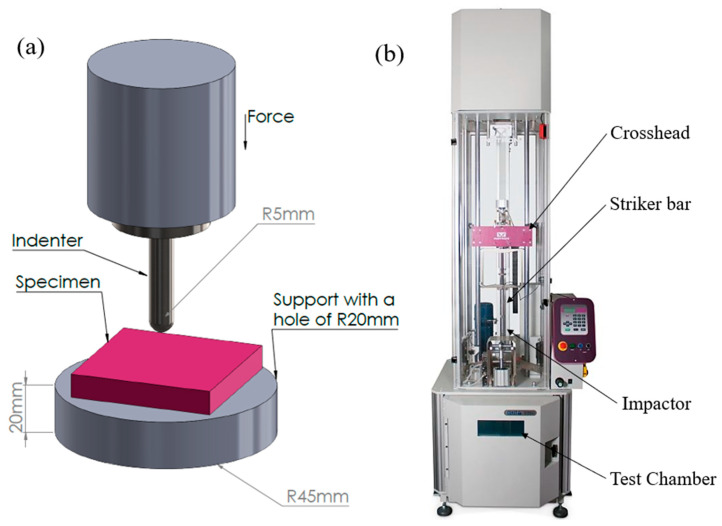
(**a**) Schematic experimental setup for quasi-static indentation (**b**) Experimental setup for the drop tower test.

The quasi-static indentation tests are performed to determine the specimens’ mechanical performance. A universal testing machine (Shimadzu, AG-X 100 kN, Kyoto, Japan) and a hemispherical indenter with a radius of 5 mm were used to conduct the quasi-static tests. Figure 4a shows that the 3D-printed constructions were set on a fixed circular support with a 40 mm diameter hole inside. Two samples were evaluated for each test condition to ensure repeatability. The indenter’s speed was maintained at 0.6 mm/min, and the maximum displacement was set to 22 mm. The maximum displacement was more than twice the specimen’s thickness for complete perforation. However, complete perforation was not observed as the loading rate was small in quasi-static indentation.

### 2.3. Drop Tower Test

The low-velocity impact performance of the specimens is assessed by the drop tower test, conducted using the Instron CEAST 9350 drop tower machine (Instron, Norwood, MA, USA). The specimens were set up in the same configuration as the quasi-static test, as shown in Figure 4a. A hemispherical impactor with a radius of 5 mm was used, and a 5 kg mass was dropped from a fixed height. The energy applied is calculated using the height and mass of the impactor. Two samples were evaluated for each test condition to ensure repeatability. The average results are then presented. The energy absorbed by the structure was calculated using Equation (1).

(1) $$E=\int_{0}^{\infty }Fd\delta$$

where *E* is the energy absorbed, *F* and δ are the applied force and displacement, respectively.

### 2.4. Scanning Electron Microscope (SEM)

**Figure 5:**
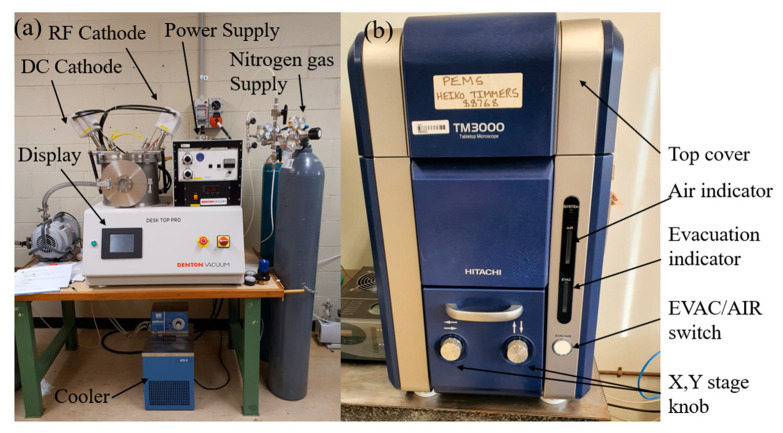
Sputtering and SEM machine; (**a**) Denton Desktop Pro; (**b**) Hitachi TM3000 microscope.

The topography of the 3D-printed surfaces was examined using an electron microscope (HITACHI TM3000, Hitachi, Tokyo, Japan) as shown in Figure 5b. The SEM images were obtained to analyze the fractured parts of the samples. The polymer parts have poor conductivity, restricting their direct use for SEM analysis. Thus, the fractured parts were coated with aluminum to get high-quality images. The coating was completed using a Denton Desktop Pro machine (Figure 5a) with a layer of about 15 nm.

### 2.5. Profilometer

**Figure 6:**
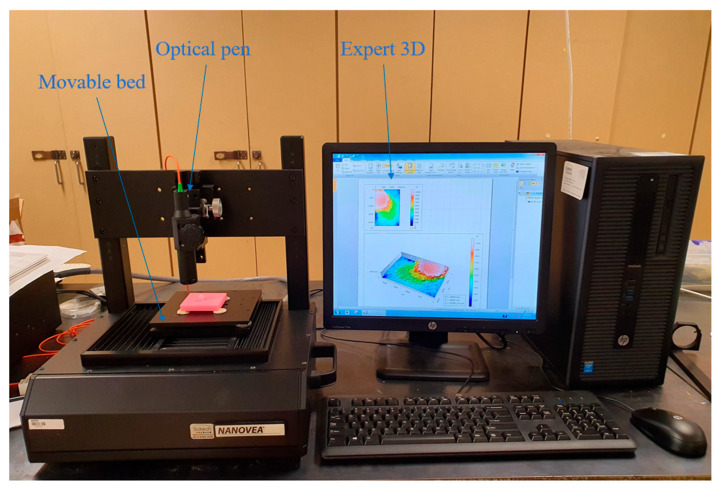
Nanovea PS50 profilometer and Expert 3D software.

A 3D non-contact profilometer (Figure 6) was used to inspect fractured surfaces. NANOVEA PS50’s optical profilometer (made by NANOVEA Inc., Irvine, CA, USA) uses the wavelength of light to calculate the actual height of the target specimen. The topographical and color representation is achieved by measuring the physical wavelength related to the vertical positions [34,35]. The micrographs of the fractured profiles were processed with 3D image software (Expert 3D, USA, version: 7.0.6966) to get the three-dimensional profile by resampling the height maps.

## 3. Results and Discussion

### 3.1. Quasi-Static Response

The infill patterns influence the mechanical response of the structures that are fabricated using additive manufacturing [36,37]. In this study, the polymer structures were fabricated in rectilinear and Bouligand patterns with an infill density of 25%, 50%, 75%, and 100%. Quasi-static tests were conducted as described in the previous section.

**Figure 7:**
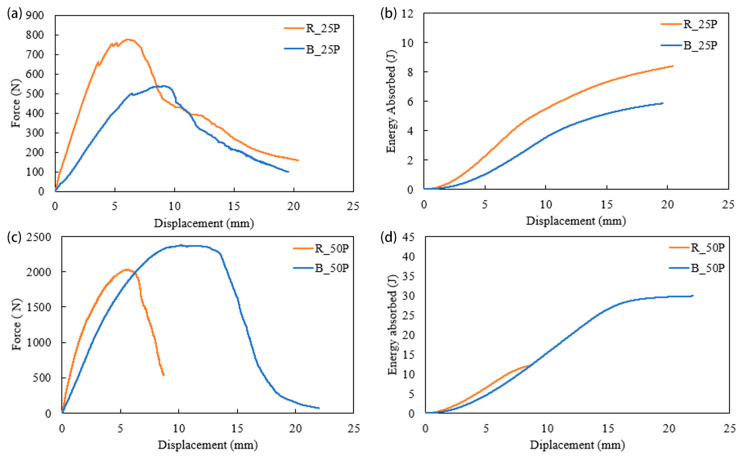

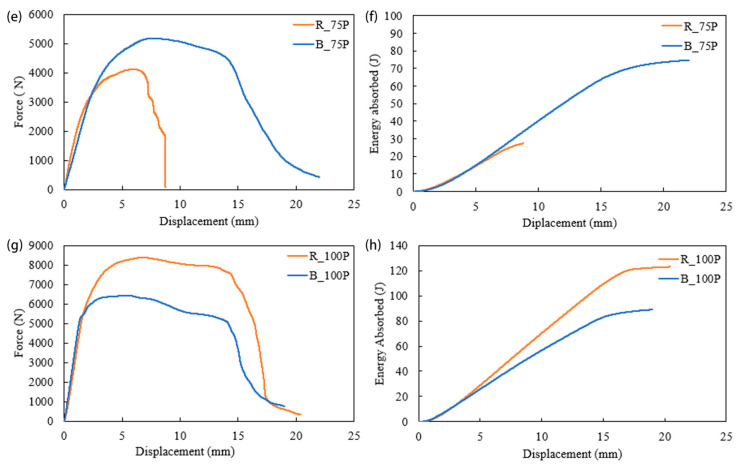
Force vs. displacement and energy absorbed vs. displacement for 25% (**a**,**b**), 50% (**c**,**d**), 75% (**e**,**f**), and 100% (**g**,**h**) infill densities under quasi-static indentation.

Figure 7 presents force, energy absorption, and displacement during quasi-static loading. The structures printed in rectilinear infill patterns with 25% and 100% infills had higher peak forces, 776 N and 8400 N, respectively (Figure 7), than their Bouligand counterparts (for 25% and 100% infills). On the other hand, Bouligand patterned structures with 50% and 75% infills exhibited higher peak forces, 2374 N and 5182 N, respectively. It is observed from the quasi-static response that the Bouligand pattern had fewer peak forces in low and high infill density. The results can be concluded from experimental results and failure analysis. The rectilinear pattern had a stiffer response in all infill densities due to its printing pattern at a 45°/−45° angle; however, as the infill density increased, it showed more crack formation along the printing angle, i.e., more brittleness. In the case of the Bouligand pattern, the layers were shifted with a 15° raster angle. The layer supporting at 25% infill density was shallow. As the infill density increased, the support for each layer was enhanced. Thus, the peak force increased by 50% and 75% infill percentages. However, no significant improvement was observed in 100% infill density as the path pattern could not be identified when the structure functioned like a solid structure.

Similar results were obtained for the energy absorption capacity of the specimens. Interestingly, the 50% and 75% infilled structures in the Bouligand pattern showed 1.25 and 1.75 times more energy absorption capacity than the rectilinear pattern. As the in-fill density increased, more layer supports were added with a 15° raster angle. Thus, large displacements were observed for 50% and 75% infill density, resulting in higher energy absorption. However, there was no significant improvement in Bouligand-patterned structures for a 100% infill density. The change in printing pattern had no impact because these acted as solid structures, and there was no porosity. The solid structures had similar cracking and failure, as discussed in Section 3.3. The peak force and energy absorption capacity increased with the structure’s infill density, which is the expected behavior for 3D-printed materials.

**Figure 8:**
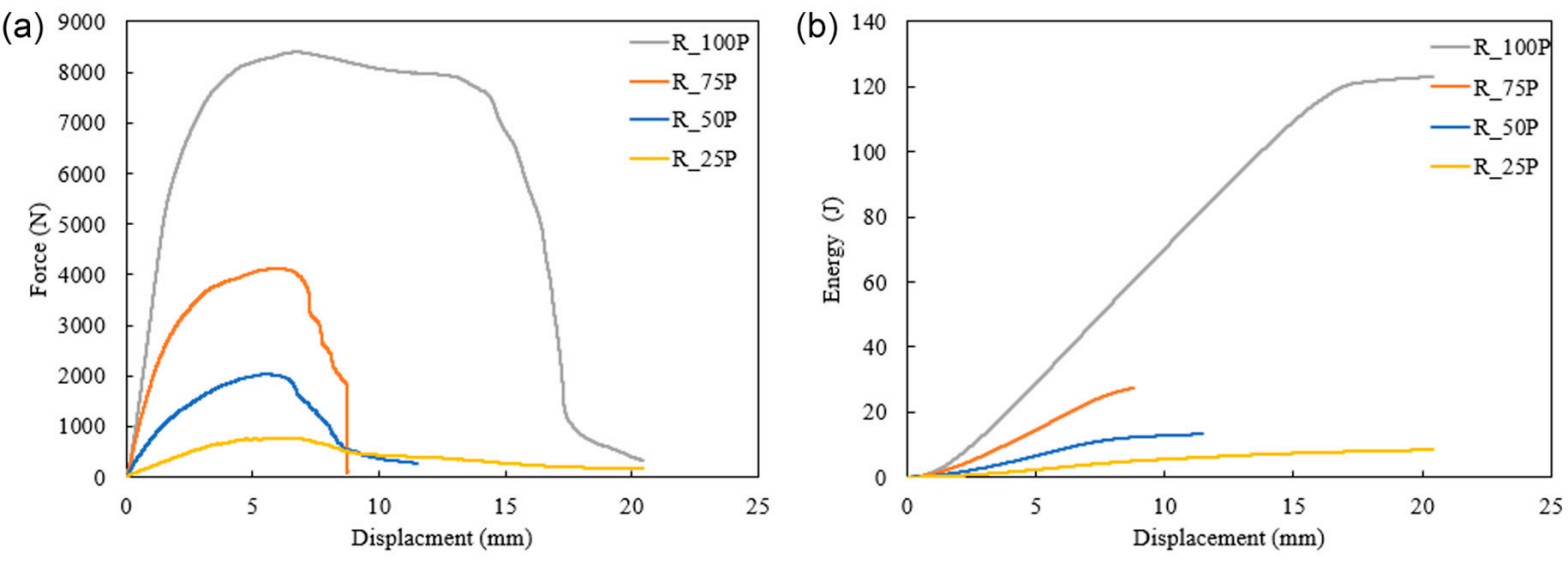
Effect of infill density in rectilinear tool path under quasi-static indentation on forece (**a**) and energe (**b**).

**Figure 9:**
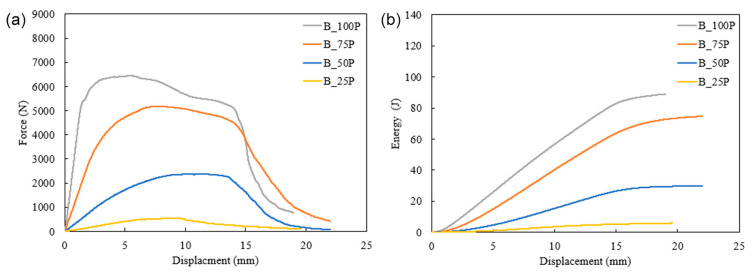
Effect of infill density in Bouligand tool path under quasi-static indentation on forece (**a**) and energe (**b**).

The difference in the infill percentage also affects the energy absorption capacity of additively manufactured specimens. The effect of infill percentage on the 3D printed structures is illustrated in Figure 8 and Figure 9. These figures present the force vs. displacement and energy vs. displacement relationships of the structures printed with four different infill densities. The highest peak force for rectilinear and Bouligand patterns is recorded in 100% infilled structures. The specimen with 75% infill fabricated in the rectilinear pattern has the most minor displacement. However, all the rectilinear patterned specimens have peak forces at around 6.5 mm displacement. It is noticed that the higher infill density showed higher energy absorption capability. The 100% infilled specimen significantly increased energy absorption compared to the other specimens, as shown in Figure 8. Specimens R_25P and R_100P had displacements that were more significant than thickness because there was hollow support in the experimental setup, as shown in Figure 4.

Specimens R_50P and R_75P had less displacement than R_25P. As the material density increased, the brittleness increased; thus, the fracture occurred abruptly. In the case of the Bouligand tool path (Figure 9), the peak force and displacement followed the same trend as the rectilinear-patterned specimens. However, the force peaks at various displacements for the Bouligand specimens. It is also noticed that the Bouligand specimens with different infill densities have similar maximum displacements. In contrast, the maximum displacement of the rectilinear specimens ranges from around 8 mm to 20 mm.

**Figure 10:**
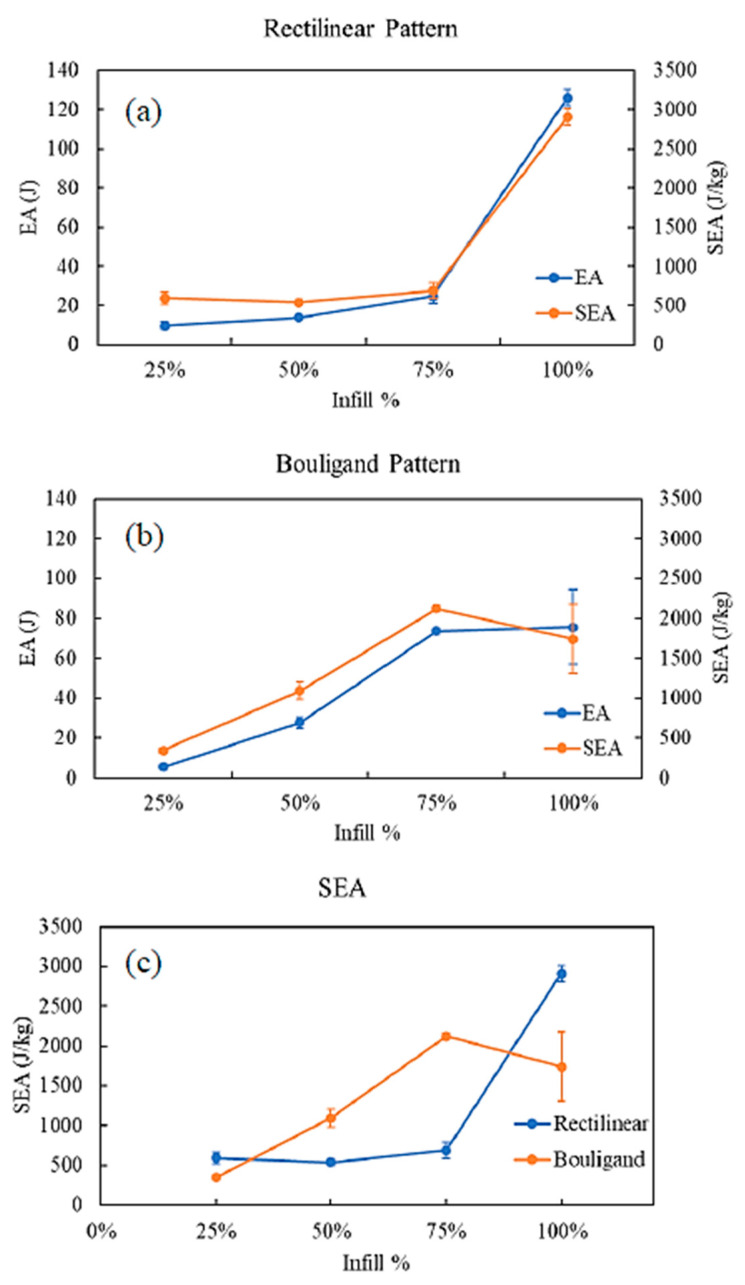
EA and SEA at different infill densities (**a**) Rectilinear, (**b**) Bouligand, (**c**) SEA comparison under quasi-static indentation.

Figure 10 shows the energy absorbed (EA) and specific energy absorption (SEA) for rectilinear and Bouligand-patterned specimens.

The samples printed at various infill densities exhibited a similar trend for both patterns. EA increased with the increase of the infill percentage. As the infill density increases, the weight of the structure also increases. Thus, getting an optimized SEA for a particular pattern is possible. SEA for 75% infilled Bouligand-patterned specimen exhibited the highest SEA compared to the other infill densities. Generally, the porous structures show better SEA than the 100% infilled structure, which is also observed in Bouligand-patterned specimens [38]. The highest SEA is 2.9 kJ/kg obtained from the 100% infilled rectilinear pattern, and the lowest SEA is 0.35 kJ/kg from the 25% infilled Bouligand pattern. It is noted that samples with 50% and 75% infills produced using the Bouligand tool path had higher SEA than those with rectilinear patterns, as illustrated in Figure 10c.

### 3.2. Low-Velocity Impact Response

**Figure 11:**
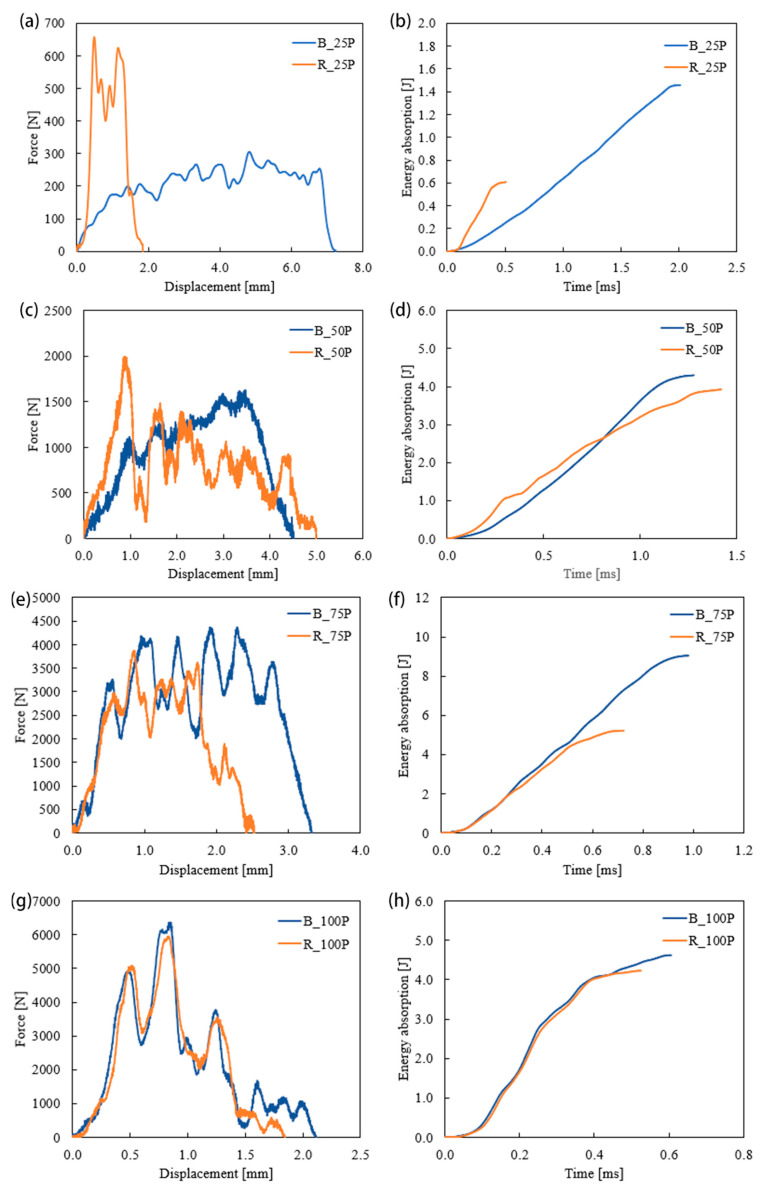
Force vs. displacement and energy absorbed vs. time for 25% (**a**,**b**), 50% (**c**,**d**), 75% (**e**,**f**), and 100% (**g**,**h**) infill densities under low-velocity impact.

The dynamic responses of the bio-inspired and rectilinear pattern specimens were examined using a drop tower setup. The effects of infill pattern and percentage were analyzed to understand the applicability of the additively manufactured specimen based on various requirements. The load vs. displacement characteristics of the Bouligand and rectilinear patterns are shown in Figure 11. Due to the low-velocity impact test process, the specimens’ absorbed energy increased until they were completely damaged. These 3D-printed polymer specimens had a brittle failure and showed no sign of rebounding like metals and fiber composites. Brittle failure behavior, including split, fracture, and perforation, was observed in the specimens.

In the case of 25% infilled specimens, R_25P exhibited a higher peak force and a shorter displacement, while B_25P had a lower force but a much longer displacement for the same thickness of the plates. This indicated that the failure time had increased due to the change of tool path.

The sudden drop of R_25P had a brittle failure with low energy absorption capacity. The elastic energy of R_25P had a higher slope than B_25P, and the crack opened around 0.5 mm. The failure occurred around 2 mm of displacement. On the other hand, the elastic energy for the B_25P was relatively low; however, the structure sustained for more extended displacement, implying ductile behavior. The area under the curve of R_25P is larger than the B_25P, which indicates R_25P had higher energy absorption up to 2 mm displacement. However, the ductile behavior of the Bouligand structure contributed to absorbing more energy until it failed. Thus, B_25P exhibited 2.5 times better energy absorption than R_25P. Similarly, B_50P absorbed more energy than R_50P. However, the R_50P specimen had taken the peak load to 2000 N, and B_50P had a lower peak force with a uniform load distribution. There was a massive improvement in the 75% infilled structures where B_75P had 1.8 times higher energy absorption than R_75P. The infill pattern had no significant effect on 100% infilled specimens. Both patterns obtained similar cracks and failures with a close impact-resistant property, as shown in Figure 11. Although the infill density increased by 100% infilled pattern specimens, they had lower impact resistance than the 75% infilled densities. Also, the Bouligand pattern exhibited better impact resistance than the rectilinear pattern in 25%, 50%, and 75% infill densities.

**Figure 12:**
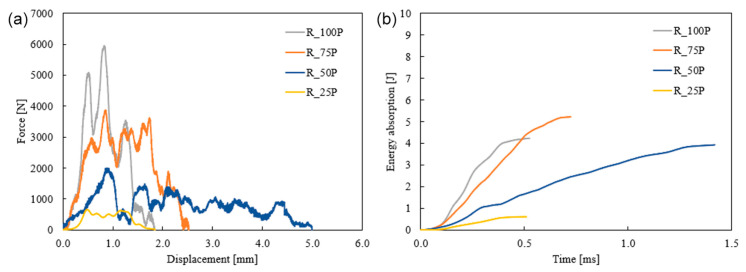
Effect of infill density for the rectilinear tool path sample on the forece (**a**) and energe (**b**) of the specimen under low-velocity impact.

The rectilinear pattern’s force and displacement response in Figure 12 showed that the peak force increased as the infill density increased. The load-carrying capacity increased as the number of printing layers increased with the infill percentages. The highest displacement was observed by 50% infilled and reduced as the porosity reduced. R_50P had more significant displacement than R_25P, whereas the displacement decreased with increased infill percentage for Bouligand samples. In the case of R_50P, the failure shows that the crack propagated to the 45° printing layers, contributing to more displacement. However, R_25P samples penetrated with much lower force and displacement have no cracks in the structure. It is noted that peak energy absorption is found in the 75% infilled specimens, whereas the 100% infilled structure has 23.4% less energy absorption capacity.

**Figure 13:**
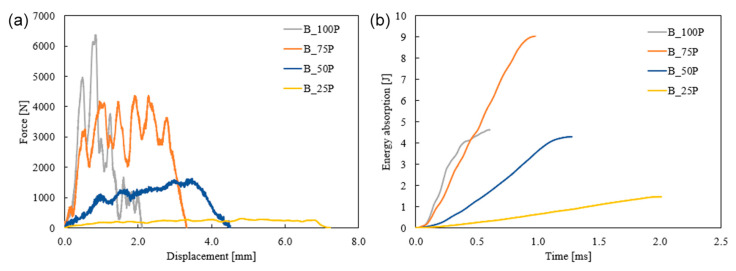
Effect of infill density for the Bouligand tool path sample on the forece (**a**) and energe (**b**) of the specimen under low-velocity impact.

Similarly, in Figure 13, the peak force for Bouligand-patterned structures increased with higher infill percentages. The most significant displacement was observed in the specimens with the highest porosity (25% infill). This occurs as porosity rises, leading to reduced crack initiation. Additionally, due to its porosity, the 75% infilled pattern exhibited better energy absorption capacity than the 100% infilled specimen. Thus, the results suggest that a 75% infilled structure is suitable for actual field applications based on the need for a porous structure with adequate energy absorption capacity.

**Figure 14:**
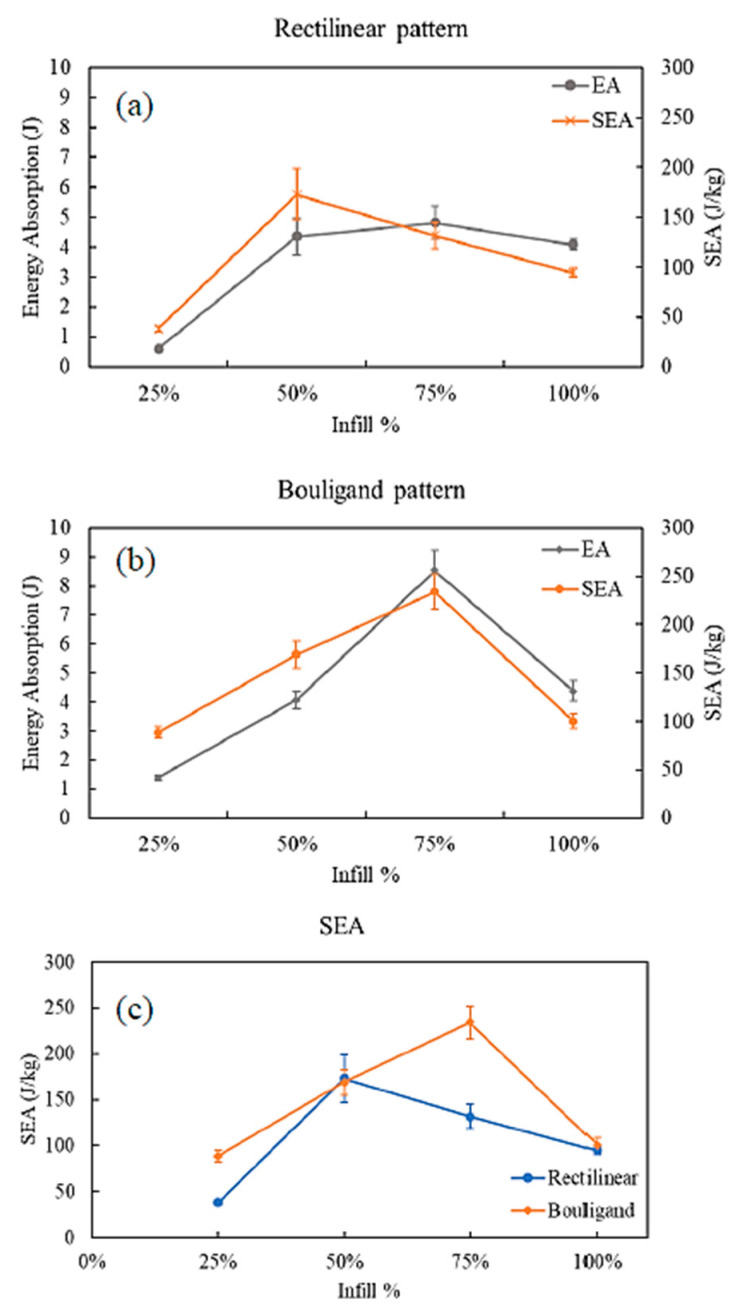
EA and SEA at different infill densities (**a**) Rectilinear, (**b**) Bouligand, (**c**) SEA comparison under low-velocity impact experiment.

As the infill percentage increases, the energy absorption rises until it reaches 75% infill density, as shown in Figure 14. It then declines, with the specimen’s porosity also decreasing. However, SEA peaked at 50% infill, while 25% infill exhibited the lowest value for the rectilinear pattern. A similar trend was observed in the EA and SEA curves for the Bouligand pattern, where both increased up to 75% infill and then decreased as density increased. The specimen with a 75% infill rate displayed the highest EA and SEA, while the specimen with a 25% infill rate had the lowest values in both cases. The Bouligand specimen consistently had a higher SEA than the rectilinear pattern across almost all infill percentages. Among all the tested specimens, the Bouligand structure with 75% infill achieves the highest SEA, whereas the rectilinear pattern with 25% infill showed the lowest SEA.

### 3.3. Fracture Analysis

#### 3.3.1. Quasi-Static Test

**Figure 15:**
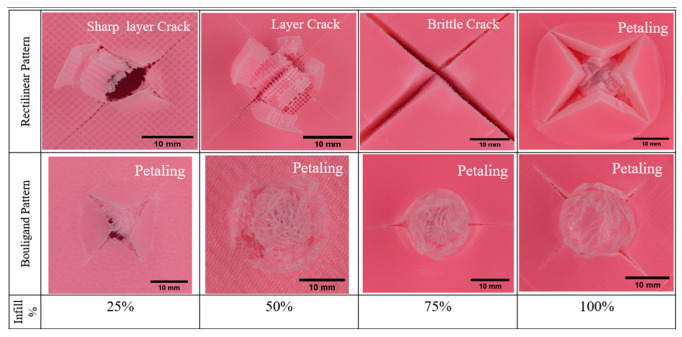
Failure morphology of the samples (back face) tested under quasi-static indentation.

The test results indicate that the load and energy-absorbing capacity varies with changes in the printing pattern. Additionally, variations in the porosity of the specimen influenced the failure morphology, as illustrated in Figure 15. It was noted that the failure area, crack formation, and damage exhibited significant differences across all specimens. The front face images of the specimens are not included here since they all display similar circular shape deformations due to the indentation. The rectilinear samples demonstrated cross-shaped cracks because the layers were arranged cross-ply. Circular shape deformation along with perforation was observed for R_25P and R_50P, with cracks propagating in the direction of the printing pattern. The R_75P sample showed large cracks at +45/−45-degree angles without damaging the filaments and broke with minimal displacement, as seen in Figure 12. Petaling damage, a large sharp crack, was noted in R_100P. This failure mode is commonly observed in thin metal and plastic plates [39].

Similar petaling damage was also observed in the samples printed with the Bouligand pattern. However, the fractured areas exhibited a textured shape deformation due to the orientation of the fused filaments. Small cracks were visible in the B_25P, B_75P, and B_100P damage, while no sharp brittle failure was detected in the Bouligand samples. The damage pattern for each sample type remained consistent across different infill densities. For instance, the shape of the crack formation was aligned with the cross-ply direction for the rectilinear pattern. In contrast, Bouligand-patterned samples displayed a textured shape deformation that became more pronounced as the infill density increased.

**Figure 16:**
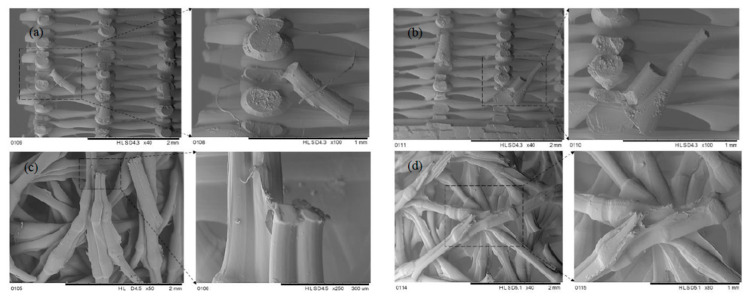
SEM images: (**a**) rectilinear pattern; (**b**) brittle fracture of the filaments; (**c**) Bouligand sample’s fracture part; (**d**) filament arrangement in the Bouligand pattern.

As illustrated in Figure 16a,b, the rectilinear pattern displayed a sharp, brittle fracture. This sharp failure contributed to an increased crack formation in the structure, leading to sudden failure. In contrast, the Bouligand pattern (Figure 16c,d) shows that the plywood arrangement of the filaments resists sudden cracking. This configuration improves the plastic behavior of the filaments, resulting in more protracted displacements observed in the Bouligand samples.

#### 3.3.2. Drop Tower Test

**Figure 17:**
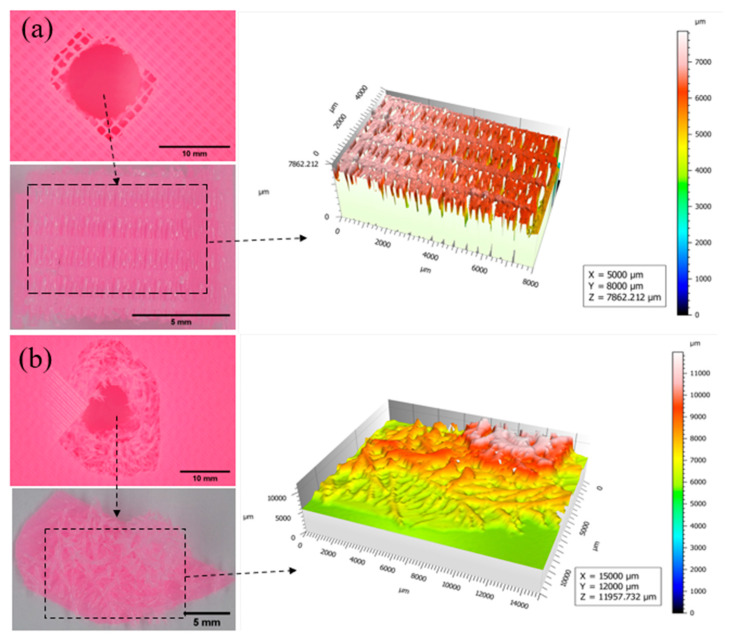
Fracture profiles of 25% infilled specimens (**a**) Rectilinear, (**b**) Bouligand.

**Figure 18:**
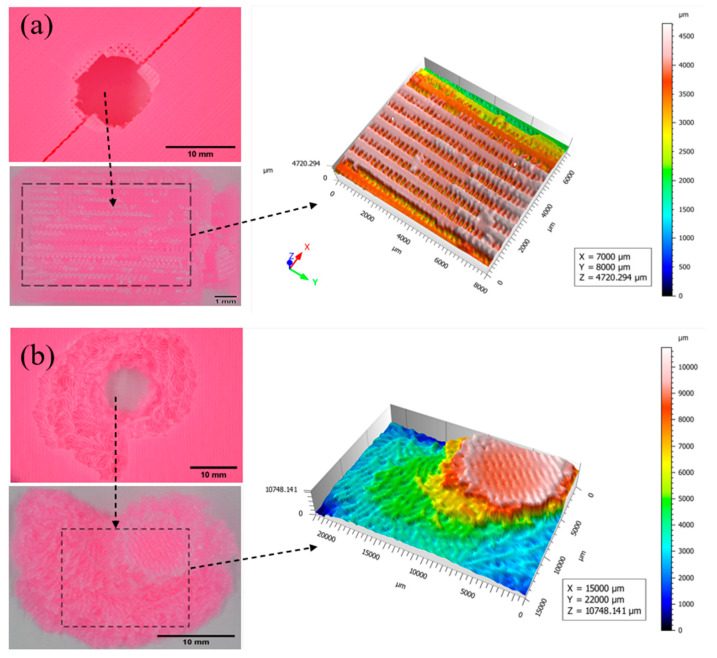
Fracture profiles of 50% infilled specimens (**a**) Rectilinear, (**b**) Bouligand.

**Figure 19:**
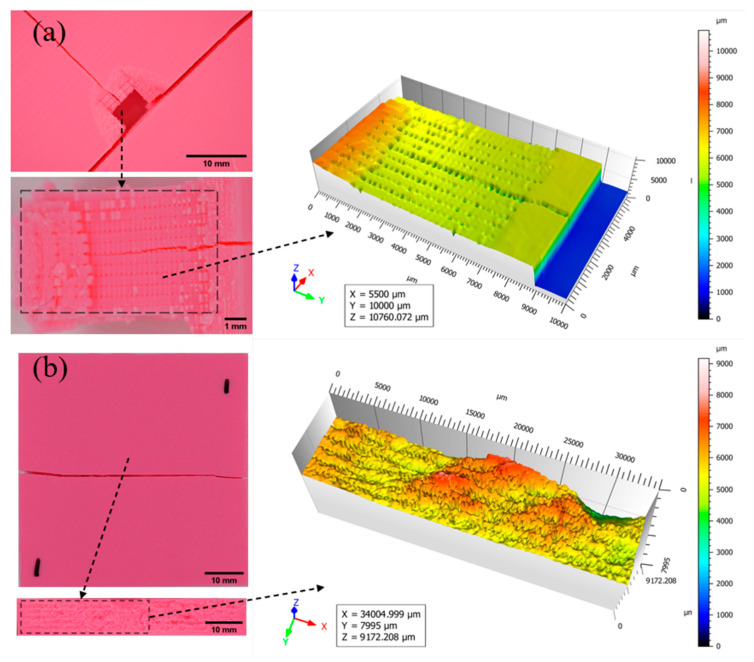
Fracture profiles of 75% infilled specimens (**a**) Rectilinear, (**b**) Bouligand.

**Figure 20:**
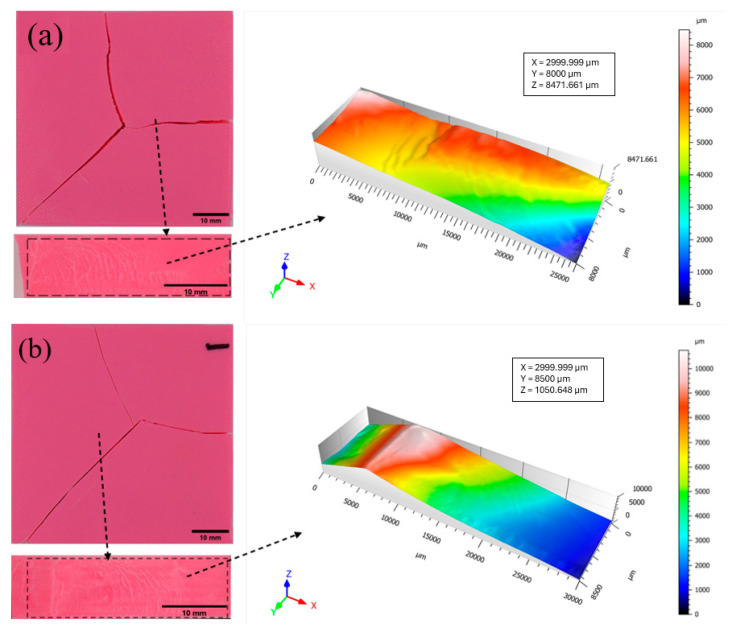
Fracture profiles of 100% infilled specimens (**a**) Rectilinear, (**b**) Bouligand.

The fracture surfaces obtained from Expert 3D (USA, version: 7.0.6966) software were examined in a relative coordinate system, where the *X* and *Y* axes reveal the surface topography, and the *Z* axis displays the height from the region’s lowest point. The values for the *X*, *Y*, and *Z* axes are presented in micrometers (µm). Surface regions were selected manually based on the fractured parts, shown in individual figures in the inset (Figure 17, Figure 18, Figure 19 and Figure 20). Perforation, splitting, and fracturing were observed in the damaged specimens. Figure 17 illustrates the fractured parts of specimens with 25% infill that have rectilinear Bouligand structures. The regular rectilinear pattern was evident in the profile of the fractured part, and the porous areas were also clearly visible. The Bouligand specimen exhibited an irregular fracture region, but the fractured filament patterns suggested a plywood-type structure where the surface heights increased in a rotational direction. A similar fracture surface is also visible in Figure 18, where the filaments were arranged more densely. The size of the fractured region of the rectilinear pattern was approximately the diameter of the impactor. In contrast, the Bouligand-patterned specimens exhibited more significant fracture regions with a plywood-like structure. It was evident that in the Bouligand-patterned structure, the fracture morphologies followed the printing pattern.

The profiles of the fractured parts of the 75% and 100% infilled specimens are illustrated in Figure 19 and Figure 20, respectively [40]. The fracture pattern changes as the infill density of the additively manufactured specimen increases. The shape of the fractured region significantly changed to rectangular as the specimen’s stiffness increased, as shown in Figure 19a. Additionally, a crack propagated along the specimen’s thickness. For the Bouligand specimen, the crack propagated in the longitudinal direction and split into two parts, as shown in Figure 19b. The tiny spikes on the cracked area revealed that the rectangular pattern failure is less ductile than the Bouligand pattern failure. Due to their 100% infill density, the fractured behaviors of both designs were highly comparable (Figure 20). Despite the multiple printing pathways, the failure mode remained unaffected. Since there are no pores, the surfaces appear relatively smooth compared to other specimens.

**Figure 21:**
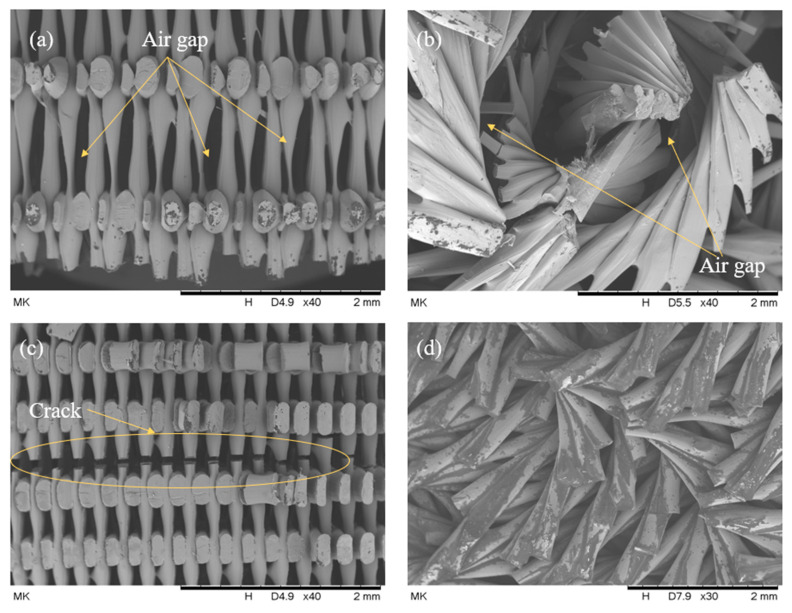
SEM topography of drop tower test samples (**a**) R_25P, (**b**) B_25P, (**c**) R_50P, and (**d**) B_50P.

Figure 21 shows the surface topography of 3D-printed samples with various infill patterns. Some fractured parts were cut into smaller pieces using an oil bath cutter. Consequently, the specimen prepared for SEM displayed visible oil traces in particular images, as seen in Figure 21d. The printing pattern and the porosity between the layers are evident in the SEM images. It was observed that the porosity in the structure decreases as the infill percentage increases, which is managed by the 3D-printing slicing software. For example, more material was visible at the 50% infill density than at the 25% infill (Figure 21). No cracks were observed between the filament arrangements, indicating high-quality printing achieved with an FDM printer.

The crack formed in Figure 21c was initiated due to the drop tower loading. This crack initiation directs how the failure of the rectilinear pattern sample propagated to the filaments. Moreover, necking-like failure was observed in the Bouligand-fractured parts, as shown in Figure 21b,d. This more significant displacement has been observed due to the Bouligand pattern’s plastic-like failure phenomenon.

## 4. Conclusions

A comparative study analyzed the quasi-static and low-velocity impact of structures printed using bio-inspired (Bouligand pattern) and traditional tool paths. The infill densities ranged from 25% to 50%, 75%, and 100% to examine the effects of infill density under various loading conditions. The study led to the following conclusions:

(i) Altering the infill percentage significantly influenced the energy absorption capacity of the additively manufactured structure. The load-carrying capacity increases as the porosity between the layers decreases.
(ii) Modifying the printing pattern can enhance the structure’s energy absorption capacity. The load-carrying capacity is also improved as the supporting layers vary due to different pattern types. In the 75% infilled case, the bio-inspired tool path offered better impact resistance than the rectilinear tool path-printed specimen.
(iii) When utilizing the Bouligand structure for designing the 3D printing tool path, the energy absorption of the specimen increased by 1.25 and 1.73 times for the 50% and 75% infills under quasi-static loading. Similarly, dynamic test results indicated that the Bouligand pattern was 1.73 times more effective in energy absorption. These findings suggest that bio-inspiration could be a viable approach to producing structures with enhanced mechanical properties.
(iv) Fracture analyses revealed that the Bouligand pattern demonstrated less brittleness than the rectilinear pattern. Consequently, the Bouligand pattern fracture exhibited more petaling failure than crack propagation. The deformed parts of dynamic failure maintained a Bouligand-like shape that mirrored the tool path, while the rectilinear pattern displayed sharp, brittle failure. This phenomenon was also clearly shown in the SEM images. However, changing the printing path did not significantly affect the specimens with no porosity (i.e., 100% infill).

This experimental setup for low-velocity impact testing has focused solely on measuring the strike velocity of the projectile using two sets of photoelectric sensors. However, future work should consider incorporating advanced technologies such as high-speed cameras or X-ray imaging to comprehensively understand and analyze failure mechanisms and target responses. By utilizing these tools, researchers can gain valuable insights into the intricate details of the impact process, capturing continuous events and visualizing the failure mechanisms within the target material. Integrating high-speed cameras or X-ray imaging into the experimental setup can enhance our understanding of impact dynamics and facilitate more accurate and detailed analyses in future studies. Certain factors for future studies could include exploring different raster angles, various sources of bio-inspiration, the effects of varying length scales, the impact of impactor velocity, and the fabrication of functionally graded structures, among others, to improve the mechanical performance of additively manufactured structures potentially. These structures have potential applications in the automotive, aerospace, and military sectors.

## Figures and Tables

**Table 1:** Physical and mechanical properties of 3D-printed PLA.

Properties	Values
Young’s modulus	3200 MPa
Ultimate strength	51.5 MPa
Flexural strength	55.2 MPa
Elongation at break	5%
Print temperature	190–210 °C
Bed temperature	None/50 °C+
Bed surface	Glass plate
Active cooling	50%
Printing speed	40–70 mm/s

**Table 2:** Printing parameters used in manufacturing specimens.

Printing Conditions	Parameters
Printing method	FDM
Material type	PLA
Average filament diameter	1.75 mm
Nozzle diameter	0.4 mm
Nozzle temperature	210 °C
Bed temperature	65 °C
Layer height	100 µm
Infill	25%, 50%, 75%, 100%
Printing speed	60 mm/s
Travel speed	80 mm/s
Infill structure	Rectilinear, Bouligand
Print orientation (rectilinear)	+45°, −45°
Print orientation (Bouligand)	0°, 15°, 30°, 45°, 60°, 75°, 90°, 105°, 120°, 135°, 150°, 165°
Bed adhesion	Brim
Slicer software	Simplify3D (version 4.3)
CAD software	Solid works (version 2021)

**Table 3:** 3D printed specimen details.

Infill Pattern	Specimen ID	Infill %	Mass (g)	Density (g/cm^3^)
Rectilinear	R_25P	25	16.45	0.46
	R_50P	50	25.24	0.70
	R_75P	75	35.74	0.99
	R_100P	100	43.29	1.20
Bouligand	B_25P	25	15.66	0.44
	B_50P	50	24.09	0.67
	B_75P	75	36.44	1.01
	B_100P	100	43.54	1.20

## Data Availability

Data will be made available upon reasonable request.

## References

[1] Chen Y., Baehr S., Turner A., Zhang Z., Gu G.X. (2021). Carbon-fiber reinforced polymer composites: A comparison of manufacturing methods on mechanical properties. Int. J. Lightweight Mater. Manuf..

[2] Isaac W., Ezekwem C. (2021). A review of the crashworthiness performance of energy absorbing composite structure within the context of materials, manufacturing and maintenance for sustainability. Compos. Struct..

[3] Ha N.S., Lu G. (2020). A review of recent research on bio-inspired structures and materials for energy absorption applications. Compos. Part B Eng..

[4] Islam M.K., Hazell P.J., Escobedo J.P., Wang H. (2021). Biomimetic armour design strategies for additive manufacturing: A review. Mater. Des..

[5] Siddique S.H., Hazell P.J., Wang H., Escobedo J.P., Ameri A.A.H. (2022). Lessons from nature: 3D printed bio-inspired porous structures for impact energy absorption—A review. Addit. Manuf..

[6] Wang C., Su D., Xie Z., Wang H., Hazell P.J., Zhang Z., Zhou M. (2022). Dynamic behaviour of Bio-inspired heterocyclic aramid Fibre-reinforced laminates subjected to Low-velocity Drop-weight impact. Compos. Part A Appl. Sci. Manuf..

[7] Dhari R.S., Patel N.P., Wang H., Hazell P.J. (2021). Numerical investigation of Fibonacci series based bio-inspired laminates under impact loading. Compos. Struct..

[8] Huang Z., Pan Z., Li H., Wei Q., Li X. (2014). Hidden energy dissipation mechanism in nacre. J. Mater. Res..

[9] Gopalan H., Chokshi A.H. (2018). The mechanical behavior of nacre across length scales. J. Mech. Behav. Biomed. Mater..

[10] Katti K.S., Katti D.R., Pradhan S.M., Bhosle A. (2011). Platelet interlocks are the key to toughness and strength in nacre. J. Mater. Res..

[11] Zhang X., Cai Z.B., Li W., Zhu M.H. (2018). Understanding hydration effects on mechanical and impacting properties of turtle shell. J. Mech. Behav. Biomed. Mater..

[12] Hu L., Sielert K., Gordon M. (2012). Turtle shell and mammal skull resistance to fracture due to predator bites and ground impact. J. Mech. Mater. Struct..

[13] Achrai B., Bar-On B., Wagner H.D. (2015). Biological armors under impact—Effect of keratin coating, and synthetic bio-inspired analogues. Bioinspir. Biomim..

[14] Szewciw L., Barthelat F. (2017). Mechanical properties of striped bass fish skin: Evidence of an exotendon function of the stratum compactum. J. Mech. Behav. Biomed. Mater..

[15] Ghods S., Murcia S., Ossa E.A., Arola D. (2019). Designed for resistance to puncture: The dynamic response of fish scales. J. Mech. Behav. Biomed. Mater..

[16] Wang L., Zhang H., Fan Y. (2011). Comparative study of the mechanical properties, micro-structure, and composition of the cranial and beak bones of the great spotted woodpecker and the lark bird. Sci. China Life Sci..

[17] Seki Y., Schneider M., Meyers M. (2005). Structure and mechanical behavior of a toucan beak. Acta Mater..

[18] Lee S., Novitskaya E.E., Reynante B., Vasquez J., Urbaniak R., Takahashi T., Woolley E., Tombolato L., Chen P.-Y., McKittrick J. (2011). Impact testing of structural biological materials. Mater. Sci. Eng. C.

[19] McKittrick J., Chen P.-Y., Bodde S.G., Yang W., Novitskaya E.E., Meyers M.A. (2012). The Structure, Functions, and Mechanical Properties of Keratin. JOM.

[20] Whitenack L.B., Simkins D.C., Motta P.J., Hirai M., Kumar A. (2010). Young’s modulus and hardness of shark tooth biomaterials. Arch. Oral Biol..

[21] Bertram J.E., Gosline J.M. (1986). Fracture toughness design in horse hoof keratin. J. Exp. Biol..

[22] Islam M.K., Wang H., Hazell P.J., Kader A., Escobedo J.P. (2022). Quasi-static response of horse hoof inspired biomimetic structures. Mater. Today Proc..

[23] Behera R.P., Le Ferrand H. (2021). Impact-resistant materials inspired by the mantis shrimp’s dactyl club. Matter.

[24] Yang F., Xie W., Meng S. (2021). Analysis and simulation of fracture behavior in naturally occurring Bouligand structures. Acta Biomater..

[25] Wang H., Wang C., Hazell P.J., Wright A., Zhang Z., Lan X., Zhang K., Zhou M. (2021). Insights into the high-velocity impact behaviour of bio-inspired composite laminates with helicoidal lay-ups. Polym. Test..

[26] Ahamed M.K., Wang H., Hazell P.J. (2022). From Biology to Biomimicry: Using Nature to Build Better Structures—A Review. Constr. Build. Mater..

[27] Podroužek J., Marcon M., Ninčević K., Wan-Wendner R. (2019). Bio-inspired 3D infill patterns for additive manufacturing and structural applications. Materials.

[28] Suksangpanya N., Yaraghi N.A., Kisailus D., Zavattieri P. (2017). Twisting cracks in Bouligand structures. J. Mech. Behav. Biomed. Mater..

[29] Guarín-Zapata N., Gomez J., Yaraghi N., Kisailus D., Zavattieri P.D. (2015). Shear wave filtering in naturally-occurring Bouligand structures. Acta Biomater..

[30] Grunenfelder L., Suksangpanya N., Salinas C., Milliron G., Yaraghi N., Herrera S., Evans-Lutterodt K., Nutt S., Zavattieri P., Kisailus D. (2014). Bio-inspired impact-resistant composites. Acta Biomater..

[31] Fazita M.R.N., Khalil H.P.S.A., Izzati A.N.A., Rizal S., Jawaid M., Thariq M., Saba N. (2019). Effects of strain rate on failure mechanisms and energy absorption in polymer composites. Failure Analysis in Biocomposites, Fibre-Reinforced Composites and Hybrid Composites.

[32] Tanveer M.Q., Haleem A., Suhaib M. (2019). Effect of variable infill density on mechanical behaviour of 3-D printed PLA specimen: An experimental investigation. SN Appl. Sci..

[33] Aloyaydi B.A., Sivasankaran S. (2020). Low-velocity impact characteristics of 3D-printed poly-lactic acid thermoplastic processed by fused deposition modeling. Trans. Indian Inst. Met..

[34] He Q., Li A., Guo Y., Liu S., Zhang Y., Kong L. (2018). Tribological properties of nanometer cerium oxide as additives in lithium grease. J. Rare Earths.

[35] Podulka P. (2022). Selection of methods of surface texture characterisation for reduction of the frequency-based errors in the measurement and data analysis processes. Sensors.

[36] Mantihal S., Prakash S., Bhandari B. (2019). Textural modification of 3D printed dark chocolate by varying internal infill structure. Food Res. Int..

[37] Pernet B., Nagel J.K., Zhang H. (2022). Compressive strength assessment of 3D printing infill patterns. Procedia CIRP.

[38] Lubombo C., Huneault M.A. (2018). Effect of infill patterns on the mechanical performance of lightweight 3D-printed cellular PLA parts. Mater. Today Commun..

[39] Wierzbicki T. (1999). Petalling of plates under explosive and impact loading. Int. J. Impact Eng..

[40] Wang K., Xie X., Wang J., Zhao A., Peng Y., Rao Y. (2020). Effects of infill characteristics and strain rate on the deformation and failure properties of additively manufactured polyamide-based composite structures. Results Phys..

